# Construction of permanently inducible miRNA-based expression vectors using site-specific recombinases

**DOI:** 10.1186/1472-6750-11-107

**Published:** 2011-11-16

**Authors:** Sara E Garwick-Coppens, Adam Herman, Scott Q Harper

**Affiliations:** 1 Center for Gene Therapy, The Research Institute at Nationwide Children's Hospital, Columbus, Ohio, 43205, USA; 2 Research Information Services, The Research Institute at Nationwide Children's Hospital, Columbus, Ohio, 43205, USA; 3 Department of Pediatrics, The Ohio State University College of Medicine, Columbus, Ohio, 43205, USA

## Abstract

**Background:**

RNA interference (RNAi) is a conserved gene silencing mechanism mediated by small inhibitory microRNAs (miRNAs).

Promoter-driven miRNA expression vectors have emerged as important tools for delivering natural or artificially designed miRNAs to eukaryotic cells and organisms. Such systems can be used to query the normal or pathogenic functions of natural miRNAs or messenger RNAs, or to therapeutically silence disease genes.

**Results:**

As with any molecular cloning procedure, building miRNA-based expression constructs requires a time investment and some molecular biology skills. To improve efficiency and accelerate the construction process, we developed a method to rapidly generate miRNA expression vectors using recombinases instead of more traditional cut-and-paste molecular cloning techniques. In addition to streamlining the construction process, our cloning strategy provides vectors with added versatility. In our system, miRNAs can be constitutively expressed from the U6 promoter, or inducibly expressed by Cre recombinase. We also engineered a built-in mechanism to destroy the vector with Flp recombinase, if desired. Finally, to further simplify the construction process, we developed a software package that automates the prediction and design of optimal miRNA sequences using our system.

**Conclusions:**

We designed and tested a modular system to rapidly clone miRNA expression cassettes. Our strategy reduces the hands-on time required to successfully generate effective constructs, and can be implemented in labs with minimal molecular cloning expertise. This versatile system provides options that permit constitutive or inducible miRNA expression, depending upon the needs of the end user. As such, it has utility for basic or translational applications.

## Background

The central dogma of molecular biology describes how the genetic information encoded in DNA is used to make proteins. Although RNAs were long known to play crucial roles in this process (e.g. mRNAs, tRNAs, rRNAs), their importance in gene expression control has dramatically expanded with the recent discovery of RNA interference (RNAi) [1,2]. In the simplest terms, RNAi refers to sequence-specific gene silencing occurring after mRNA transcription, and the principal regulators of this process are small, non-coding microRNAs (miRNAs) [3,4].

Natural miRNAs are encoded in the genomes of eukaryotes and some viruses [2-7]. Over 1,000 miRNAs were identified in recent years, and the major steps and players involved in miRNA biogenesis have been largely defined [8-24]. This knowledge provided the foundation for developing expression systems to deliver engineered inhibitory RNAs, like shRNAs and artificial miRNAs, to various cells and tissues *in vitro *and *in vivo *[9,25-28]. In essence, these vectors operate as siRNA or mature miRNA delivery vehicles; as there is not universal nomenclature for such systems, we note that they are referred to as "artificial miRNA shuttles" in this manuscript. Importantly, these artificial miRNA shuttles can be engineered to knockdown any gene of interest [9,25,26,28,29]. As a result, they have emerged as powerful molecular tools for querying natural miRNA function, delivering inhibitory RNAs for functional genomics studies, or developing RNAi-based therapies for disease [30]. Indeed, several pre-clinical studies support that RNAi-based therapies are promising approaches for treating dominant genetic diseases, viral pathogens, and cancer [30-40].

**Figure 1:**
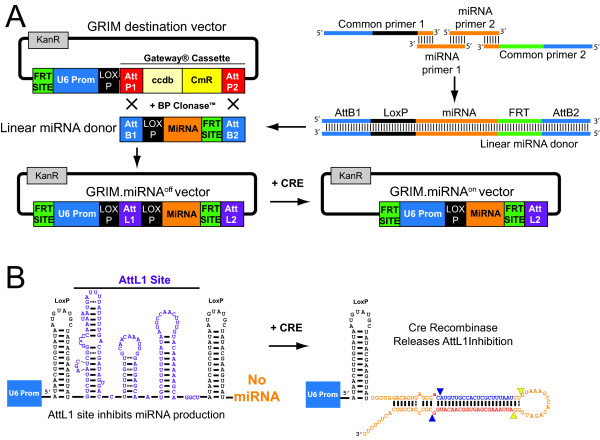
**Overview of GRIM system**. Details are described in the Results and Methods sections. (A) GRIM.miRNA vectors are constructed by BP recombination between a linear miRNA donor and our GRIM destination vector. Each linear miRNA donor is created by extending 4 DNA primers. Common primers 1 and 2 are used to build every linear miRNA donor DNA, and contain identical AttB, LoxP, and FRT sequences, as shown. The middle portion of the linear miRNA donor is generated by DNA oligos containing miRNA sequences designed by the end-user or using our GRIM.REAPER software package (Additional File 4). BP recombination produces the GRIM.miRNA^off ^vector, which contains a LoxP-flanked 5' AttL1 site that inhibits miRNA production. The AttL1 site can be removed by Cre recombinase to produce the constitutively active GRIM.miRNA^on ^vector. The entire U6 promoter (U6 prom) miRNA cassette is flanked by FRT sites, which can be utilized to shut down the system in the presence of Flp recombinase. (B) Predicted secondary structure of the LoxP-flanked AttL1 site, which inhibits miRNA production. Cre excision of AttL1 releases this inhibition, thereby permitting miRNA expression. The miLacZ sequence is shown here (blue indicates sense strand; red indicates antisense guide strand). Nucleotides in orange are stem and loop sequences derived from human miR-30a, except the 3' terminal poly U, which is an engineered termination signal for the pol III-dependent U6 promoter. The blue and yellow arrowheads indicate Drosha and Dicer cut sites, respectively.

Our laboratory is interested in developing RNAi therapies for dominantly inherited disorders [34,40]. This work requires delivering artificial therapeutic miRNA shuttles to target cells and tissues. Numerous groups, including ours, have described various expression vectors in recent years [25-28,32,33,36,41-51]. The most commonly used systems rely upon constitutively active promoters, such as U6, to drive inhibitory RNA transcription. These vectors are typically constructed using traditional cut-and-paste molecular cloning techniques, and multiple constructs are often required to identify ones capable of effectively silencing target genes [29]. To streamline the construction process and make it less labor-intensive, we developed a procedure to rapidly generate artificial miRNA expression vectors using a commercially available phage recombinase system (Gateway^® ^Technology from Invitrogen™). In so doing, we also created a method that provides tightly controlled, inducible miRNA expression, and a built-in mechanism to destroy the vector, if desired. We named our method the Gateway^®^-Ready Inducible MiRNA (GRIM) expression system (Figure 1). In this manuscript, we describe a detailed protocol for GRIM vector construction and demonstrate the functionality of our system in mammalian cells.

## Methods

### GRIM Destination Vector construction

The GRIM destination vector was constructed by recombinant PCR from two products. The first product contained the mouse U6 promoter flanked by single FRT and LOXP sites at the 5' and 3' ends, respectively. In addition, 18 nucleotides corresponding to the 5' portion of the AttP1 site were added downstream of LOXP (product A). Product A primers were: 577 Forward (5' TTCAATTGTAGACTAGTGAAGTTCCTATTCTCTAGAAAG 3') and 685 Reverse (5' GGGGCCCGAGCTTAAGACATAACTTCGTAT AGCATACATTATAC 3'). The second product (Product B) was the Gateway^® ^Cassette, containing the ccdB and chloramphenicol resistance (CmR) genes flanked by AttP1 and AttP2 sites, which was amplified from pDONR221. This cassette was also engineered to contain 25 nucleotides of a LoxP site at the 5' end. Product B primers were: 684 Forward (5' GTATAATGTATGCTATACGAAGTTATGTCTTAAGCTCGGGCCCC 3') and 578 Reverse (5' AACAATTGGTAACATCAGAGATTTTGAGACAC 3'). Products A and B were then stitched together by 10 cycles of recombinant PCR using the overlapping LoxP and AttP1 sequences, followed by 30 cycles of exponential amplification using primers 577 and 578. The resultant 2,922 bp PCR product was gel purified, cloned into pCR^®^-Blunt II-TOPO (Invitrogen), transformed into One Shot^® ^ccdB Survival™ competent cells (Invitrogen), and plated onto LB agar plates containing chloramphenicol. Minipreps from restriction positive colonies were sequenced to confirm accuracy.

### Linear miRNA Donor construction

The 219 bp linear miRNA donor sequences were constructed from 4 primers, two of which are common to all constructs, and two that are unique to the cloned miRNA sequences. This strategy is described in the Results section and primer sequences detailed in Additional File 1. For this study, we built GRIM vectors containing two different microRNA sequences (miLacZ and miGFP) targeting LacZ and eGFP reporters, respectively (Additional File 1). For miRNA donor construction, one microgram of each primer was mixed together in a 50 microliter reaction with Pfx polymerase (Invitrogen) and thermocycled using the following conditions: 95°C × 30 sec, 45°× 30 sec, and 68°C × 30 sec for 35 cycles. Full-length miRNA donor bands were visualized on ethidium bromide stained agarose/TBE gels (Additional File 2) to ensure primer extension. However, we found that gel or column purification was unnecessary for BP cloning into the GRIM destination vector.

### BP recombination reaction and clone verification

Three microliters of the 50-microliter miRNA donor reaction was combined with 150 ng of the Gateway^® ^Destination vector, and this DNA mixture was then added to a 10 microliter, room temperature BP Clonase reaction (Invitrogen) for 1 hour using manufacturer's instructions. BP Clonase was inactivated with Proteinase K, following manufacturer's instructions, and 2 microliters of the BP reaction was transformed into chemically competent, ccdB sensitive TOP10 *E. coli *cells (Invitrogen). Colonies were selected on LB agar plates containing kanamycin. Resistant colonies were DNA miniprepped (Qiagen Spin miniprep kit) and digested with EcoRI (New England Biolabs), and electrophoresed on a 1% agarose/TBE/ethidium bromide gel to determine clone correctness (Additional File 2). Correctly recombined clones (GRIM.miRNA^off^) show two EcoRI bands of 3,501 and 900 bp, while the empty GRIM destination vector has three EcoRI fragments of 3,501, 2,170, and 805 bp (Additional File 2). BP recombination had a 90% efficiency for both the miLacZ and miGFP constructs. Restriction positive colonies were DNA sequenced (Big Dye^® ^Terminator Cycle Sequencing Kit, Applied Biosystems) using a primer located at the 3' end of the U6 promoter (5' CACAAAAGGA AACTCACCC 3').

### Generating GRIM.miRNA^on ^vectors in *E. coli*

GRIM.miRNA^off ^vectors can be permanently switched on by transformation into EL350 *E. coli *cells, which express Cre recombinase from the arabinose-inducible promoter [52]. Cre recombinase was induced by L-(+)-Arabinose prior to making cells electrocompetent. Cre^+ ^EL350 cells were then transformed with 1 ng GRIM.miRNA^off ^plasmid, and grown overnight at 32°C on LB agar plates containing kanamycin. Resistant colonies were seeded into miniprep cultures, and agitated overnight in LB/kanamycin liquid media at 32°C. Following DNA miniprep, FLOXed clones were identified by EcoRI digestion and electrophoresis. Positive GRIM.miRNA^on ^colonies show two EcoRI bands at 3,501 and 725 bp, compared to 3,501 and 900 bp from unFLOXed GRIM.miRNA^off ^vectors (Additional File 3). In our experiments, Cre excision was 100% efficient in EL350 cells.

### Transient gene silencing studies

#### GRIM.miGFP

**Figure 2:**
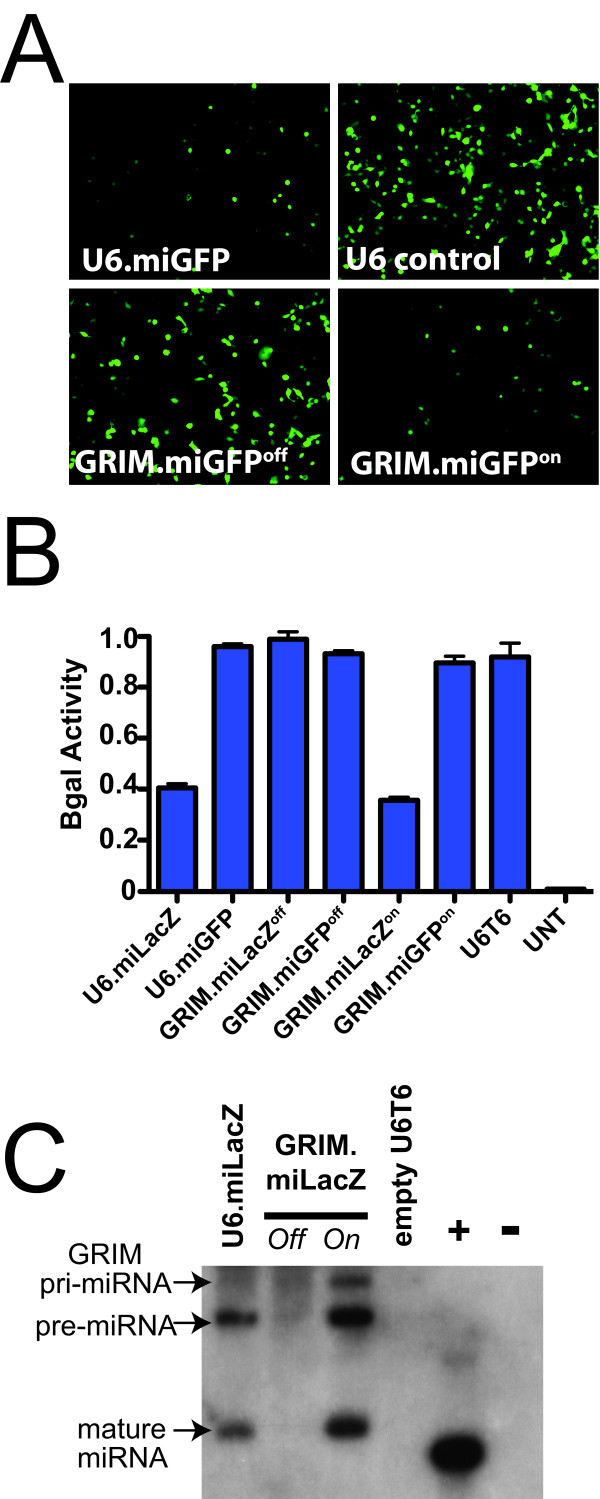
**GRIM.miRNA^off ^vectors are turned 'on' by Cre recombinase**. (A) GFP epifluorescence in HEK293 cells demonstrates GRIM.miGFP^on ^functionality. Cells were transfected 24 hours prior with CMV.eGFP and indicated miRNA expression plasmids. GFP knockdown was evident in cells transfected with the traditionally cloned U6.miGFP plasmid or its GRIM.miGFP^on ^counterpart, compared to cells containing an empty U6 promoter (U6 control). In contrast, GRIM.miGFP^off ^did not cause GFP gene silencing. Images shown are representative of three independent experiments. (B) βgal assay confirms the Cre-inducibility of the GRIM system. HEK293 cells were transfected with CMV.LacZ and indicated miRNA or control expression plasmids. Only the traditionally cloned U6.miLacZ and the GRIM.miLacZ^on ^analog caused statistically significant LacZ gene silencing (ANOVA, p < 0.05). All other constructs had no impact on βgal activity. Error bars indicate standard error of the mean (s.e.m.). U6T6 is an empty U6 promoter plasmid. UNT, untransfected HEK293 cells. (C) Small transcript northern blot shows that Cre induces full-length and processed GRIM.miLacZ. In contrast, GRIM.miLacZ^off ^vectors do not express full-length or processed miRNAs. +, indicates a positive control DNA oligo containing the miLacZ antisense sequences. -, indicates negative DNA control corresponding to the miLacZ sense strand. A radiolabeled version of this oligo was also used to probe the blot.

HEK293 cells were co-transfected (Lipofectamine-2000, Invitrogen) with 25 ng of a CMV.eGFP expression vector (pVETLeGFP)[41] and 775 ng indicated miRNA expression plasmid (Figure 2) in duplicate on 24 well plates. GFP epifluorescence was imaged using a fluorescent inverted microscope equipped with a digital camera 24 hours later.

GRIM.miL*acZ*. HEK293 cells were co-transfected with 20 ng CMV.βgal expression plasmid and 180 ng of the indicated miRNA expression constructs (Figure 2) in triplicate on 96 well plates with white walls and clear bottoms. The next day, cells were lysed for 10 minutes using 10 microliters of Galacto-Light™ kit lysis buffer (Applied Biosystems). One microliter of lysate was used to quantify total protein by Lowry assay (BioRad DC™ Protein Assay). β-galactosidase activity was then measured from the remaining 9 microliters of lysate following manufacturer's instructions. Triplicate data were averaged per condition, and three independent experiments performed. β-galactosidase activity was normalized to Lowry assay-determined protein content.

#### GRIM.miLacZ with Flp/FRT system

**Figure 3:**
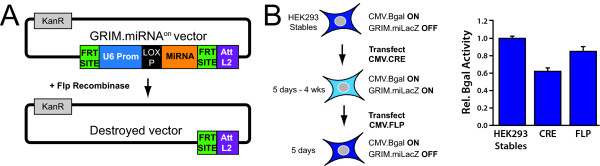
**GRIM.miRNA^on ^vectors are turned 'off' by Flp recombinase**. (A) GRIM.miRNA^on ^vectors contain FRT sites flanking the U6.miRNA sequences. The entire U6.miRNA expression cassette can therefore be removed with Flp recombinase, thereby permanently switching the vector 'off'. (B and C) Testing the Flp off-switch. We generated a line of HEK293 cells stably expressing a CMV.βgal the GRIM.miLacZ^off ^plasmids (described in Methods). Transient transfection of CMV.Cre significantly reduced βgal activity in these cells, and this silencing was maintained for up to 4 weeks (our longest timepoint) compared to the parent, unFLOXed cells (ANOVA, p < 0.05). βgal activity was significantly increased 5 days after transiently transfecting GRIM.miLacZ^on ^stable cells with the pOG44 CMV.Flp expression plasmid (ANOVA, p < 0.05), which encodes a Flp protein isoform with suboptimal activity at 37°C. Error bars indicate s.e.m.

HEK293 cells were co-transfected with 10 ng CMV.βgal and indicated plasmids (Figure 3B). Where indicated, cells received 30 ng CMV.Cre, 130 ng GRIM.miLacZoff, and/or 30 ng CMV.Flp recombinase. βgal activity was measured as described above. All wells received equivalent molar amounts of plasmid, such that an empty U6 plasmid was used to normalize DNA loading in transfection liposomes, when necessary.

### Northern blot

Four micrograms of indicated GRIM.miLacZ and control plasmids were transfected into HEK293 cells on 6 well plates. One day later, total RNA was extracted using the miRVANA kit (Ambion). Five micrograms of total RNA per well was loaded on an urea/polyacrylamide/TBE gel, along with DNA oligonucleotides containing antisense (+) or sense (-) miLacZ sequences, as previously described [33]. The gel was run at 20 mA until bromophenol blue dye reached the bottom, then separated RNAs were transferred to Nylon membranes (Nytran N+) at 200 mA for 45 minutes in 0.5× TBE. Membranes were then cross-linked and hybridized with a ^32^P end-labelled DNA oligo (5'-End Labelling Kit, GE Healthcare) that detected the antisense guide strand of the miLacZ sequence, in Oligo Hyb Buffer (Ambion) for two days at 36°C. The membrane was then washed three times at room temperature and twice at 36°C in 2× SSC, exposed to autoradiographic film (Amersham Hyperfilm™, GE Healthcare) at -80°C, and developed 7 days later.

### Generation of Stable HEK293 cells

HEK293 cells were transfected (Lipofectamine-2000, Invitrogen) with a 1:3 molar ratio of CMV.βgal and PGK.Neo plasmids in a 60 mm plate. Two days later, 400 μg/ml G418 was added to tissue culture media (DMEM with 10% FBS, 1% penicillin/streptomycin, 1% L-glutamine) and cells were passaged and selected for 4 weeks. G418 resistant cells were then plated at low density, 24 different single colonies were picked and expanded, and representative cells from each colony were stained for β-gal activity (β-gal Staining Kit, Invitrogen) using manufacturer's instructions. Six of 24 colonies were βgal positive, and we ultimately selected a line that had consistent βgal expression in all cells. This line was then co-transfected (Lipofectamine-2000) in a 12-well plate with 1280 ng GRIM.miLacZ^off ^and 320 ng of a CAG.Puromycin Resistance (PuroR) plasmid. Two days later, we began selection in Puromycin (1 μg/ml concentration in media) for one month, and then PCR screened resistant cells for GRIM.miLacZ^off ^presence.

### Validation of the Flp/FRT System in Stable HEK293/βgal cells

Puro^R^/G418^R ^stable HEK293/βgal/GRIM.miLacZ^off ^cells were transiently transfected with a CMV.Cre expression plasmid (Lipofectamine-2000, Invitrogen), and βgal activity was determined in the parent and FLOXed stable cells 5 days later, to ensure GRIM.miLacZ-mediated βgal knockdown. Four weeks later, FLOXed GRIM.miLacZ^on ^cells were transfected with the pOG44 CMV.Flp expression plasmid (Stratagene). βgal activity was then measured 5 days later using the Galacto-Light™ kit lysis buffer (Applied Biosystems), as described above. Data are representative of 3 independent experiments.

## Results

### Construction of GRIM vectors: Overview

Any inhibitory RNA sequence can be cloned into the GRIM vector system using our universal strategy. A general overview of this method is shown in Figure 1 and the detailed protocol is described in Additional Files 1,2 and 3. The initial cloning reaction requires three major components: the GRIM destination vector, a linear miRNA donor, and BP Clonase™ enzyme, derived from lambda phage recombinases (Invitrogen™).

The GRIM destination vector contains the RNA polymerase III-dependent mouse U6 promoter cloned upstream of a Gateway^® ^selection cassette. Single FRT and LoxP sites, which are substrates for Flp and Cre recombinases, respectively, flank the U6 promoter at its 5' and 3' ends (Figure 1A).

In our system, the linear miRNA donor is based on sequences and structures derived from human miR-30a (Additional File 1) [9,17,28]. We build the miRNA donor by annealing 4 DNA primers and filling in gaps with a thermostable DNA polymerase, such as Taq or Pfx. The two outside primers (Figure 1A; common primers 1 and 2) are universal for building all linear miRNA donors, while the two internal ones (miRNA primers 1 and 2) provide the unique miRNA sequences designed by the end user (Figure 1A and Additional File 1). Common primer 1 contains a terminal AttB1 site followed by a LoxP site and 15 nucleotides derived from the lower stem of human miR-30a (Figure 1 and Additional File 1). Common primer 2 is a minus strand oligonucleotide encoding a terminal AttB2 site, an optional FRT site, 13 nucleotides of the human miR-30a lower stem, and a RNA polymerase III termination signal (TTTTTT). In addition to encoding the unique miRNA sense and antisense sequences, the internal miRNA primers 1 and 2 also contain human miR-30a stem and loop sequences, which provide common complementary regions for primer annealing. The primer design and annealing strategy is detailed in Additional File 1. In addition, we developed a software package that automates the GRIM.miRNA shuttle prediction and design process (Additional File 4).

Extending the two common and two miRNA-specific primers produces a full-length 219 bp linear miRNA donor DNA (Additional Files 1 and 2), which is then used in a BP recombination reaction with the GRIM destination vector (Figure 1). Smaller, incompletely extended products are also generated but lack full-length AttB sites and are therefore incapable of participating in the BP reaction (Additional File 2). As a result, gel purification of the full-length linear miRNA donor DNA is unnecessary (discussed in greater detail in supplementary materials; Additional Files 1, 2 and 3).

The parent GRIM destination vector is kanamycin resistant (KanR) and contains an active *ccdB (control of cell death B) *gene in the Gateway^® ^cassette region. *CcdB *gene expression is lethal to most transformation-competent *E. coli *used in laboratories for molecular cloning purposes. BP recombination replaces the *ccdB*-containing Gateway^® ^cassette with the linear miRNA donor DNA. This reaction also converts AttB and AttP sites to a 118 bp hybrid sequence called AttL (Figure 1). Thus, *ccdB*-sensitive cells will only grow when transformed with properly recombined plasmids, which we termed GRIM.miRNA^off ^vectors.

GRIM.miRNA^off ^plasmids have KanR and contain a LoxP-flanked AttL1 site located between the mouse U6 promoter and the artificial miRNA shuttle sequence (Figure 1B). This entire cassette is enclosed at both ends by FRT sites. The AttL1 site inhibits miRNA production, thereby conferring inducibility to the system. Mature, functional miRNAs are therefore only produced after the AttL1 site is excised (a.k.a. FLOXed) by Cre recombinase, yielding GRIM.miRNA^on ^vectors (Figure 1B, Additional File 3). Alternatively, to rapidly generate un-inducible miRNA vectors, GRIM.miRNA^off ^vectors can be transformed into EL350 *E. coli*, which express arabinose-inducible Cre recombinase (Additional File 3). Finally, the flanking FRT sites permit irreversible GRIM.miRNA^on ^shutdown in the presence of Flp recombinase (Figure 1A).

### GRIM.miRNA^off ^vectors are turned 'on' by Cre recombinase

To demonstrate the functionality of our system, we developed two different miRNA donors targeting the *E. coli *LacZ and *A. victoria *enhanced GFP (eGFP) genes, respectively (Additional File 1, Figure 2). We called these sequences GRIM.miLacZ and GRIM.miGFP. We first tested the gene-silencing efficacy of each construct relative to a comparable U6.miRNA vector prepared using conventional cut-and-paste cloning techniques (U6.miGFP and U6.miLacZ). To do this, we co-transfected HEK293 cells with a CMV.eGFP expression plasmid and U6.miGFP, an empty U6 vector, GRIM.miGFP^off^, or GRIM.miGFP^on ^constructs. Fluorescent microscopic imaging 24 hours later revealed distinctly less GFP expression in cells transfected with the U6.miGFP and GRIM.miGFP^on ^vectors compared to the empty U6 control. In contrast, GFP levels were comparably higher and more widespread in cells expressing the GRIM.miGFP^off ^plasmid (Figure 2A).

To better quantify gene silencing and confirm that the functionality of our system was not restricted to the miGFP sequence alone, we performed a second gene silencing experiment using LacZ-directed miRNAs (miLacZ) and a β-galactosidase (β-gal) reporter assay. Specifically, we transiently co-transfected HEK293 cells with a CMV.LacZ plasmid and conventional U6 or GRIM-driven miLacZ and control expression vectors (Figure 2B). One day later, we determined miLacZ-directed gene silencing by measuring β-gal activity in transfected cells. We found that cells expressing U6.miLacZ and its GRIM.miLacZ^on ^counterpart had a statistically significant 60% reduction in β-gal activity compared to all other controls (Figure 2B). In contrast, the GRIM.miLacZ^off ^plasmid had no impact on β-gal levels (Figure 2B).

Our gene silencing results using both GFP and LacZ reporter assays suggested that the unFLOXed GRIM.miLacZ^off ^vectors were incapable of producing mature, functional miLacZ sequences. To determine this, we performed small transcript northern blots using RNA isolated from HEK293 cells transfected with empty U6 vector, U6.miLacZ, and the FLOXed and unFLOXed GRIM.miLacZ constructs. The original U6.miRNA cassettes and their GRIM.miRNA^on ^counterparts are identical sequences except the GRIM vectors contain a single LoxP at the 5' end of the miRNA shuttle transcript, and are therefore 34 nt longer (Figure 1B). Using a radiolabeled probe targeting the miLacZ antisense strand, we detected identical pre-miLacZ and mature miLacZ species in U6.miLacZ and GRIM.miLacZ^on ^samples, but not in the empty U6 control or, importantly, the GRIM.miLacZ^off ^lanes (Figure 2C and Additional File 1). Moreover, the GRIM.miLacZ^on ^sample had an additional, larger band that migrated at the expected location of the LoxP-containing primary GRIM transcript (Figure 1B and Figure 2C). These results demonstrated that GRIM vectors are tightly regulated by Cre recombinase.

### GRIM.miRNA vectors can be turned 'off' by Flp recombinase

In some cases, it may be desirable to shutdown miRNA expression. For example, over-expressed inhibitory RNAs can sometimes cause unintended, non-specific off-target effects that could be detrimental to the host cell [33,53-55]. Alternatively, strategies for querying normal miRNA function could include measuring specific outcomes in miRNA-expressing cells and then determining how these outcomes change when the miRNA is turned off. We therefore built a mechanism into the GRIM system to permanently remove the FRT-flanked miRNA expression cassette with Flp recombinase (Figure 3).

We hypothesized that the optimal method to demonstrate off-switch functionality was to reverse, rather than prevent, GRIM.miLacZ-mediated βgal gene silencing. This strategy required turning 'on' the GRIM.miLacZ^off ^vector and allowing sufficient time for βgal reduction before shutting down the system with Flp. We hypothesized that this could not be accomplished in the 2-3 day time window provided by a transient transfection experiment. We therefore generated stable HEK293 cell lines expressing G418-selected CMV.LacZ and Puromycin-selected GRIM.miLacZ^off ^plasmids. After selection, we transfected HEK293 stables with CMV.Cre, cultured cells for several weeks, transfected again with CMV.Flp, and then harvested cells for βgal assays. As expected Cre-treated cells (GRIM.miLacZ^on^) had significantly reduced βgal activity compared to HEK293 stables expressing GRIM.miLacZ^off^. Importantly, transient Flp recombinase expression significantly reversed GRIM.miLacZ^on ^gene silencing (Figure 3B). These results confirmed that the GRIM system could be inactivated by Flp recombinase.

## Discussion

Numerous inhibitory RNA expression vectors have been described in recent years [25,26,28,29,36,42-51]. First-generation vector systems typically relied upon constitutively active, RNA polymerase III-dependent (pol III) promoters, such as H1 or U6, to drive uncontrolled inhibitory RNA expression [25,29,32]. As the applicability of RNAi as a technology became more apparent, additional expression strategies emerged, including embedding miRNAs into introns or 3' UTRs of translated genes, and using constitutively active or tissue-specific RNA polymerase II-dependent (pol II) promoters to direct transcription [36,41]. To achieve even greater control of inhibitory RNA expression, several groups developed doxycycline- or Cre recombinase-inducible shRNA/miRNA systems [42-49]. These inducible vectors have proven useful for studying normal miRNA function, functional genomics, and may also have potential therapeutic applications, by providing fail-safe mechanisms to shut down inhibitory RNA expression if unintended off-target effects arise.

Despite the diversity of expression strategies currently available, virtually all share the common feature that construction requires cut-and-paste molecular techniques, which can be tedious and time-consuming. In addition, developing optimal artificial inhibitory RNAs, such as for therapeutic purposes, often requires building and screening numerous constructs, which adds to the hands-on time needed to ultimately identify the best sequences [29]. Thus, our initial goal for engineering the GRIM system was to simply streamline the construction process by using a procedure dependent upon recombinases instead of restriction enzymes and DNA ligase. We decided to use the Gateway system because it was a proven method, and the enzyme cocktail required to implement the strategy was commercially available (BP Clonase by Invitrogen).

As shown in Figure 1, our GRIM strategy incorporates AttB sites onto the 5' and 3' ends of a linear DNA template for a mir-30-based artificial miRNA shuttle. These AttB sites can then recombine with AttP sites located in the GRIM destination vector, to produce a longer AttL site, which is an AttB/AttP hybrid (BP recombination; Figure 1B). During the conceptual stages of this method, we modelled the GRIM.miRNA^off ^transcript *in silico*; based on its sequence and structure, we hypothesized that the AttL site could interfere with miRNA production because of steric hindrance and/or premature transcriptional termination. Specifically, the AttL sequence was predicted to fold into multiple hairpin secondary structures (Figure 1B) that could inhibit transcriptional read-through to the miRNA, and/or impair miRNA processing (steric hindrance); and the AttL DNA template contained three stretches of termination signals for pol III-dependent transcripts (4 or more T's; Figure 1B and Additional File 5), which could prevent transcription of the full-length primary miRNA transcript (premature termination). From these concerns arose the strategy to FLOX the AttL site and remove it with Cre recombinase. Although we predicted the AttL site would partially interfere with miRNA expression, we were surprised to discover that its presence prevented miRNA production altogether (Figures 1, 2). As a result, Cre-inducibility emerged as an important, albeit somewhat unexpected, feature of the GRIM system. Nevertheless, the GRIM system is versatile, and Cre-inducibility is optional. As previously mentioned, our primary intention was to accelerate the miRNA shuttle construction process, and our initial strategy involved removing the inhibitory AttL site by simple transformation of GRIM.miRNA^off ^plasmids into Cre-expressing, EL350 *E. coli*. This virtually foolproof step produces a GRIM.miRNA^on ^plasmid that is then constitutively 'on' when delivered to mammalian cells.

We also built in a mechanism to excise the GRIM.miRNA^on ^cassette and turn it 'off' permanently with Flp recombinase, and tested the functionality of this off-switch in stable cell lines (Figure 3). Our goal here was to demonstrate that Flp recombinase could negatively regulate our system. Indeed, transient Flp expression significantly reversed GRIM.miLacZ-mediated silencing, but we noted that βgal activity did not return to baseline (Figure 3). This was likely because the heat-labile CMV.Flp construct we used (pOG44, Stratagene) has low activity at 37°C [56], which was the growth temperature of our HEK293 stables. Indeed, the optimal temperature for pOG44-derived Flp is 30°C. Exposing GRIM.miLacZ^on ^stable HEK293 cells to heat-labile Flp for a longer period of time, or expressing a thermostable form of the protein, would further improve the efficiency of gene silencing reversal.

Finally, the current GRIM vector described here relies on the RNA pol III-dependent U6 promoter to drive miRNA shuttle transcription. However, our strategy is likely not limited to pol III promoters alone. Indeed, inserting pol II poly A signals (AATAAA) in two different locations within the current GRIM system would enable the use of tissue-specific pol II promoters (Additional File 5). First, the pol III termination signal (TTTTTT) placed at the 3' end of the artificial miRNA can be replaced by one or more minimal pol II poly A signals (AATAAA). Second, if the putative pol III termination signals in the AttL site inhibit miRNA production via premature termination of the primary transcript, an analogous situation can be produced for a pol II promoter-driven transcript by simply placing one or more AATAAA signals in the GRIM destination vector just downstream of the first LoxP site (Additional File 5). Cre excision would then de-repress the pol II-dependent GRIM vector, allowing processive miRNA transcription until correct termination at the downstream polyA signal. As with the pol III-based system described in this manuscript, the option to build in the Flp/FRT shutdown mechanism would also provide the vector with additional versatility. Thus, although the functionality of such a system remains to be tested, building tissue-specific pol II promoters into the GRIM system could further expand the utility of this technology.

## Conclusions

We developed a simple, rapid, and versatile system to generate miRNA expression plasmids using site-specific recombinases. One of the major advantages of our strategy is ease of construction; this feature has benefits for labs with extensive or minimal molecular biology skills (Additional File 6). In addition, our method has a built-in Cre recombinase-dependent 'on' switch that permits tight control of expression, and an optional mechanism to turn expression 'off' using Flp recombinase. Our system can therefore be used for more traditional gene silencing experiments, but also has applications for making inducible transgenic animals or RNAi therapeutics [44,57-60].

## Authors' contributions

SQH and SEGC designed the GRIM cloning strategy and wrote the manuscript. SEGC cloned and experimentally tested all vectors. SQH performed northern blots. AH and SQH designed the GRIM.miRNA prediction software package. All authors read and approved the final manuscript.

## Supplementary Material

Additional file 1 **Detailed strategy for designing artificial GRIM miRNAs and the primers required for their construction**. *Step1*. This strategy can be used to express a natural miRNA. However, in this protocol, we describe engineering artificial GRIM miRNAs for knocking down any gene of interest. Each GRIM miRNA is based on hsa-miR-30a sequences and structure. The natural mir-30a mature sequences are replaced by unique sense (blue text) and antisense (red text) sequences derived from the target gene. In this example, we show the miLacZ and miGFP constructs used in this manuscript. The orange nucleotides are derived from human miR-30a, except the 3' terminal poly U, which is added for use as a termination signal for the pol III-dependent U6 promoter. The natural mir-30 Drosha and Dicer cut sites are maintained and indicated by blue and yellow arrowheads, respectively. The mismatch located just upstream of the Drosha cut site (at position -2) should be maintained for proper processing. Choosing effective artificial miRNA sequences targeting a gene of interest still requires some trial-and-error empirical testing. However, there are several publications describing specific rules for optimal inhibitory RNA design. We found that incorporating two such guidelines improves the chances of identifying effective artificial miRNA sequences. First, cellular gene silencing proteins (RISC, RNA induced silencing complex) preferentially load the more thermodynamically unstable 5' end of a miRNA duplex (i.e. more AU-rich). Thus, to ensure proper loading of the antisense guide strand, the antisense miRNA strand should have more GC base pairs at its 3' end than its 5' end (i.e. the last 3-4 bases on either side; see LacZ and GFP target sites as an example of this requirement). Second, the mature miRNA sequence should have 60% or less GC content. To simplify design, we built both "rules" into our GRIM.REAPER miRNA design program (Additional File 4). *Step 2*. The GRIM.miRNA is ultimately transcribed from a DNA template. Step 2 illustrates the specific primer extension strategy used to create the DNA template for a GRIM.miRNA. Four primers are annealed and extended using a thermostable DNA polymerase, such as Taq or Pfx. Commons Primers 1 and 2 are used for all GRIM.miRNA constructions. Two unique primers with some common features are used to generate each specific miRNA, as shown in the miLacZ and miGFP examples here. Although the design strategy provided here permits the end-user to create miRNAs and primer sequences manually, we have automated this process using the GRIM.REAPER design program (Additional File 4). Click here for file

Additional file 2 **Generating GRIM.miRNA vectors and screening for positive clones. *Step 3***. Producing the DNA template for a Gateway Ready miRNA by thermocycling 4 DNA oligos. One microgram each of Common primers 1 and 2, and unique miRNA primers 1 and 2 (generated by the GRIM.REAPER design program), were annealed and extended in a 50 microliter Pfx polymerase reaction (Invitrogen) as follows: 95°C × 30 sec, 45°× 30 sec, and 68°C × 30 sec for 35 cycles. The full-length 219 bp miRNA donor DNA was visualized on an ethidium bromide stained agarose gel. BP recombination of this product does not require gel or column purification. *Step 4: *BP Reaction. As described in the Methods section, BP recombination results in loss of the *ccdB/CmR *cassette in the GRIM destination vector. This permits propagation in *ccdB*-sensitive *E. coli *(i.e. most competent cell strains like DH5α or TOP10). Positive colonies are also kanamycin resistant. Correctly recombined GRIM.miRNA^off ^plasmids have two EcoRI bands of 900 bp and 3,501 bp, as shown. In our hands, 90% of *ccdB *negative/kanamycin resistant colonies showed the correct EcoRI restriction pattern. *Step 5: *DNA sequencing. Plasmids showing the correct EcoRI restriction pattern are DNA sequenced using an oligonucleotide primer located at the 3' end of the U6 promoter (5' CACAAAAGGAAACTCACCC 3'). Click here for file

Additional file 3 **Regulating the GRIM system with Cre or Flp. *Step 6***. The GRIM.miRNA**^off ^**vector is turned 'on' by Cre recombinase excision of the inhibitory AttL1 site. To make a constitutively active vector, AttL1 excision can be accomplished through transformation of the GRIM.miRNA**^off ^**vector into EL350 ***E. Coli***, which express Cre recombinase. This 100% efficient reaction removes 175 bp from the plasmid, such that EcoRI digestion yields 725 bp and 3,501 bp bands, as shown. Alternatively, GRIM.miRNA**^off ^**vectors can be inducibly turned 'on' in animal cells or tissues expressing Cre recombinase from tissue- or temporal-specific promoters. *Step 7*. The GRIM.miRNA^on ^vector can be turned 'off' with Flp recombinase, as described in the Methods. Click here for file

Additional file 4 **GRIM.REAPER miRNA design program**. Using the criteria described in Additional File 1, this Java-based software package predicts highly functional artificial miRNAs from gene sequences entered by the end-user. In addition, it outputs the sequence of the DNA oligonucleotides required to build each GRIM.miRNA construct. Instructions: (1) Paste the DNA sequence of the gene of interest into the GRIM.REAPER query box. (2) Save the output text file locally. (3) Open the file. Example output: Query sequence: 1073 nt ---------------------- miRNA position: 70-91 Mature Duplex: 5' CGUUUGGACCCCGAGCCAAACU 3' |||||||||||||||||||| 3' GAGCAAACCUGGGGCUCGGUUU 5' Full length GRIM.miRNA sequence: 5'UGUUGGACAGUGAGCGAUCGUUUGGACCCCGAGCCAAACUGUAAAGCCACAGAUGGGUUUGGCUCGGGGUCCA
AACGAGCGCCUACUGUCACUUUUUU 3' miRNA primer 1: 5' CCCATCTGTGGCTTTACAGTTTGGCTCGGGGTCCAAACGATCGCTCACTGTCCAAC 3' miRNA primer 2: 5' CTGTAAAGCCACAGATGGGTTTGGCTCGGGGTCCAAACGAGCGCCTACTGTCACTTT 3'. Click here for file

Additional file 5 **Model for converting the GRIM system from pol III to pol II dependence**. (A) We hypothesize that RNA pol III termination signals (4-6 T's) in the inhibitory AttL1 site cause premature termination of GRIM.miRNAs, which prevents miRNA maturation and ultimately, gene silencing. Cre removes the AttL1-resident poly T terminators, thereby permitting full-length GRIM miRNA transcription and maturation. (B) Based on this model, the current GRIM system could be used with a pol II-based promoter as well, with some modifications. Specifically, the GRIM destination vector can be modified to contain a pol II responsive poly A signal downstream of the 5' LoxP site, which would serve to prematurely terminate a full-length GRIM.miRNA. A second poly A signal, downstream of the miRNA cassette, can then be added during the miRNA donor construction. In the unFLOXed state, the upstream poly A signal would be utilized thereby preventing miRNA production and subsequent gene silencing. Cre-mediated excision (FLOXed state) would remove the upstream poly A signal along with the AttL1 site, thereby permitting full-length miRNA transcription, maturation, and gene silencing. Click here for file

Additional file 6 **Flow chart comparing traditional cut-and-paste molecular cloning methods versus the GRIM system**. This chart illustrates the streamlined cloning process afforded by the GRIM system, compared to more traditional molecular construction methods. GRIM cloning requires markedly less hands-on and overall time for miRNA expression vector construction. Indeed, correct clones can be generated and confirmed 1-2 days earlier than with traditional molecular methods. Moreover, restriction enzyme and ligation-based methods can sometimes be inefficient and vector generation may require repeated attempts. The high cloning efficiency of the GRIM system (~90%) circumvents this problem. Click here for file
